# Supplementary material for: Using knowledge translation to support the use of evaluation findings: A case study of the linda mama free maternity program in Kenya
Source: PLOS Glob Public Health. 2024 Dec 12;4(12):e0003961. doi: 10.1371/journal.pgph.0003961 (PMC11637383; doi:10.1371/journal.pgph.0003961)
Supplement: S1 Table — (DOCX) [file pgph.0003961.s002.docx]

**Using knowledge translation to support the use of evaluation findings: A case study of the Linda Mama Free Maternity Program in Kenya**

Fatuma H Guleid^1*^, Stacey Orangi^1^, Angela Kairu^1^, Brian Arwa^1^, Janet Keru^2^, Anne Musuva^2^, Ileana Vilcu^3^, Anooj Pattnaik^4^, Nirmala Ravishankar^4^, Edwine Barasa^1,5^

^1^ Health Economics Research Unit (HERU), KEMRI-Wellcome Trust Research programme

^2^ ThinkWell, Nairobi, Kenya

^3^ Thinkwell, Geneva, Switzerland

^4^ ThinkWell, Washington DC, USA

^5^ Centre for Tropical Medicine and Global Health, Nuffield Department of Medicine, University of Oxford, Oxford, UK

*Corresponding author

Email: fguleid@kemri-wellcome.org

S1 Table: Progress and perceived outcomes of implementing action plans in the counties.

| **Identified challenge** | **Actionable solution** | **Progress** | **Perceived outcomes** |
| --- | --- | --- | --- |
|  | **County A** | | |
|  | **County officials (CDOH)** | | |
| Inadequate communication between CDoH, NHIF and providers | Host county health forum involving key stakeholders to disseminate information on NHIF schemes | Forums were postponed due to COVID-19 restrictions. Officials opted for smaller meetings with NHIF and other stakeholders. | The number of LM claims had increased in 2 county A sub-county facilities. In addition, NHIF collections had also doubled at the county referral hospital. |
| Inadequate staff capacity development | Train key technical staff on: - registration, claims, disbursements, and reconciliation of Linda Mama (LM) and other NHIF schemes. | Facility staff were trained on LM claims processes and re-training was ongoing. The county dedicated 2 staff members to specifically process LM claims in some facilities |  |
| Lack of supportive legal framework | Lobby for facility autonomy support revenue collection, banking at facility bank accounts | A regulation was passed by the county assembly to support revenue collection at facility level. |  |
|  | **NHIF officials** | | |
| Inadequate communication between NHIF and facilities | Carry out re-sensitisation of providers on LM during other NHIF outreach program | Started monthly meetings with healthcare facilities to sensitise them on LM benefits and processes | The participants reported that NHIF had built stronger relationships with the county government through improved working relationships. NHIF also noticed that the number of facilities that were now claiming LM benefits had increased although the increase was modest. |
|  | Attend quarterly meetings with County Government officials | With support from the development partners, NHIF now has regular meetings with the county officials |  |
| Limited LM benefit package | Communication to  MoH on the limitation of LM benefit package | This was yet to be implemented |  |
|  | Integration of the benefit package with UHC specifically to ensure that it includes Level 2 (dispensaries) and 3 (health centers) facilities | This was yet to be implemented |  |
| Documentation challenges in processing LM claims | Refresher training for county providers | This was implemented at facility level |  |
|  | Facilities to be provided with ICT equipment to facilitate processing of claims | This was identified as a responsibility for the county government to follow-up and ensure facilities budgeted/were provided with the necessary equipment |  |
|  | **Health providers** | | |
| Inadequate communication with the public, NHIF and CDOH | Conduct community dialogue days and school health talks to inform mothers on LM | Some facilities conducted the dialogue days. All facilities mentioned that they sensitise mothers through health talks or one on one at the facilities and also rely on community health volunteers (CHVs) who sensitise the mothers on LM. | The number of facilities claiming for LM benefits was reported to have increased. Additionally, there was increased registration to the program and utilisation of services by mothers. |
|  | Document facility challenges for escalation to CDOH | This was implemented in some facilities but was not regular. |  |
|  | Sensitise communities on teenage pregnancies and the LM services available to them through CHVs | CHVs are actively involved in sensitising the community about LM but interview participants were not sure if that was specific to teenage pregnancies too. |  |
| Teenage pregnancies | Improve facility friendliness to teenage mothers & targeted sensitisation to expectant teenagers | Some facilities indicated friendliness to teenage mothers was improved. This was done by forming support groups for teenage mothers and training staff on how to manage teenage pregnancies. |  |
| Inadequate claims processing | Appoint NHIF focal persons in the facilities where feasible | Facilities had appointed some staff members as the focal NHIF persons. The staff were trained by NHIF. |  |

| **Problem** | **Actionable solution** | **Progress** | **Perceived outcomes** |
| --- | --- | --- | --- |
|  | **County B** | | |
|  | **County officials (CDOH)** | | |
| Inadequate information on LM (all other NHIF schemes) shared with providers | Regular sensitisation on LM (and other NHIF schemes) of county and Sub-county health facilities | County officials sensitised facilities on the need to do claims. Designated accountants were assigned to every subcounty to assist in processing claims. Finally, county officials requested all facilities to appoint someone to handle registration, reconciliation and reporting of LM. | Interview participants reported that claims process had become smoother and with more facilities claiming benefits, reimbursements had increased. |
| Inadequate communication between NHIF and county | County officials were to host quarterly meetings with NHIF focal persons to update on any issues | Two meetings with NHIF took place as physical meetings were difficult. County officials were notified of the facilities that claim for LM and the gaps with claiming |  |
| Leakages of revenue collection | Request that all facilities submit LM/ all financial reports to the sub-county health management team (SCHMT) | Submission to the SCHMT is happening. Quarterly summary reports are also submitted. |  |
|  | **NHIF officials** | | |
| Inadequate communication with the public on LM | Carry out re-sensitisation of providers on LM during other NHIF outreach program | NHIF had outreach programs (including radio shows) targeting the public and educated the public about LM and other schemes. For facility sensitisation, facilities sent a representative to NHIF for training. | Interview participants noted that claims process had improved and the number of rejected claims due to poor documentation is decreasing. |
|  | Conduct quarterly meetings with County government officials | While 2 meetings have taken place, they have not been regular. |  |
| Limited LM benefit package | Communicate to  MoH on limitation of LM benefit package | The participant was not up to date with the progress on this. |  |
|  | Encourage women (who can afford) to enroll for Supa cover scheme as a supplement to LM | This was implemented during the outreach programs with the public |  |
| Documentation challenges in processing LM claims | Conduct refresher trainings for county providers | This was currently ongoing |  |
|  | Facilities to be provided with ICT equipment to facilitate processing of claims | This was identified as an action point for the county government |  |
|  | **Health providers** | | |
| Inadequate sensitisation of the public on LM | Conduct health talks to inform mothers on LM | All facilities mentioned that they sensitise mothers through health talks or one on one conversation at the facilities and also rely on CHVs who sensitise the mothers. | The number of facilities claiming for LM benefits increased. There was also increased registration to the program and utilisation of services by mothers. |
|  | Extend maternity open days to reach all expectant mothers | This was implemented in some facilities but was not regular. |  |
|  | Sensitise communities on LM through CHV | This is ongoing. CHVs are actively involved in sensitising the community about LM |  |
|  | Use videography and posters to spread awareness of LM | Some facilities had posters and signage about LM and NHIF schemes |  |
| Inadequate training/capacity of staff on LM claims processing | Engage NHIF in regular meetings | This was yet to be implemented |  |
|  | Invite NHIF during in-charge meetings to sensitise the staff | This was yet to be implemented |  |
|  | Conduct a training needs assessment for staff | This was done in one facility |  |
|  | Training more than one staff in the facility on claims processing | This was done in some facilities but not in others where only clerks were trained on LM |  |
| Lack of computer, printers, copiers etc | Level 2/3 facilities should budget for hardware such as printers. | Facilities received printers however some noted that computers were also needed |  |
| Stigma of teenage pregnancies | Facilities to improve responsiveness of services for teenage pregnancies | One facility noted that they had a youth center that supports services for teenage mothers. In addition, all facilities mentioned that they provide more privacy to teenager mothers and separate them from older mothers |  |
|  | Engage with the community on teenage pregnancies | This is ongoing through the health talks. In addition, community health volunteers play a vital role in spreading awareness within the communities. |  |

| **Problem** | **Actionable solution** | **Progress** | **Perceived outcomes** |
| --- | --- | --- | --- |
|  | **County C** | | |
|  | **County officials (CDOH)** | | |
| Low level of processed LM claims by subcounty facilities | Arrange frequent updates with NHIF on health facilities' progress through monthly meetings | Updates were provided. NHIF paid visits to facilities in the county. However, this was not sustained. | More facilities are claiming LM benefits, which has increased reimbursements to facilities. |
|  | Coordinate with NHIF to ensure claims are processed weekly in each sub county | This was implemented in only 2 sub-counties |  |
|  | Disseminate a circular from the county chief officer emphasising the need to increase revenue collection in all public health facilities regularly | It has not been done formally, however, participants mentioned that it is set as an agenda and discussed at their meetings. |  |
| Lack of communication flow from CDOH, NHIF to providers | Facilitate training of providers on LM and other NHIF schemes | Providers were encouraged to visit NHIF offices for sensitisation and training on claims submissions |  |
|  | Include NHIF officials in county health facilities' meeting | NHIF attend meetings when invited by county officials |  |
| Inadequate quality management | Institutionalise quality management visits at county and sub county facilities | The county conducts quarterly support visits. However, it was difficult to track impact due to lack of resources for follow up |  |
| Non-visibility of revenues at health facility level by sub-county and county providers | Develop regular reports on revenue collected | Monthly reporting on revenue streams including LM to the CDOH is now required, especially for level 4 facilities |  |
|  | Develop dashboards on revenue collection and spending | This is yet to be implemented |  |
|  | **NHIF officials** | | |
| Inadequate communication with CDOH, providers and the public | Conduct re-sensitisation of providers on LM during other NHIF outreach programs | NHIF attends county meetings with all facility in-charges. At these meetings, in-charges are sensitised on LM and other NHIF schemes. | Claims submission had improved due to training and thus processing took a shorter time. More facilities have started claiming LM benefits |
|  | Attend quarterly meetings with County government officials | NHIF have been meeting with the county director of planning at the county department of health. This has not occurred on a quarterly basis but only as needed. |  |
| Limited Benefit package | Communicate to  MoH on the limitations of LM benefit package | There has been some engagement with MoH but there have been no outcomes yet. |  |
| Documentation challenges in LM claims process | Carry out refresher training for county providers | This was currently ongoing. Providers are also trained on the online claims system |  |
|  | Facilities to be provided with ICT equipment to facilitate processing of claims | This was identified as an action point for the county government |  |
|  | **Health providers** | | |
|  | Conduct health talks to inform mothers on LM | All participants noted that they held health talks at the facilities | The number of facilities claiming for LM benefits increased. There was increased registration to the program and utilisation of services by mothers. |
| Poor sensitisation of mothers | Maternity open days to be extended to accommodate more expectant mothers | This was implemented in all facilities but was not regular due to limited funds. |  |
|  | Sensitise communities on LM through community strategies | This is ongoing. Facilities also arrange outreach programs with health promotion officers and CHVs |  |
|  | Ensure that the health promotion officer incorporates LM in health talks | Facilities had health promotion officers who participated in health talks to the mothers. In the facility without an officer, they made a request to have one appointed. |  |
| Documentation of LM claims challenges | Ensure that the NHIF focal persons in facilities should have a checklist on documentation requirements | All NHIF focal persons were provided a checklist in all the facilities to aid processing of claims |  |
| Inadequate hardware and delays in lodging claims | Hire focal NHIF clerks to lodge claims on behalf of level2/3 stationed at the subcounty office | All facilities included had hired NHIF clerks |  |
|  | Allocate money each quarter for purchasing of ICT hardware | All facilities budgeted for ICT hardware to support claims submissions |  |
|  | Use mobile phones for LM registration/notification and sensitisation on the same | Only one facility reported not having mobile phones. |  |
|  | Track lodged and paid LM claims at facility level | The new NHIF clerks were tasked with tracking claims |  |
| Stigma of teenage pregnancies | Strengthen adolescent health education | Participants mentioned that this occurred during the health talks hosted by the facilities. |  |
|  | Establishing youth friendly services in the facilities | In one facility, they had dedicated a new building unit for youth services. However, during COVID-19, this unit was used as a COVID-19 unit. |  |
|  | Host community dialogue days to address stigma of teenage pregnancies | Only one facility had hosted one community dialogue day |  |
|  | Sensitise of health workers on stigma of teenage pregnancies | This is ongoing and is done during facility meetings with in-charges. |  |
