# Supplementary material for: Using knowledge translation to support the use of evaluation findings: A case study of the linda mama free maternity program in Kenya
Source: PLOS Glob Public Health. 2024 Dec 12;4(12):e0003961. doi: 10.1371/journal.pgph.0003961 (PMC11637383; doi:10.1371/journal.pgph.0003961)
Supplement: Attachment — Submitted filename: 27022024_Response to reviewers_PLOS.docx [file pgph.0003961.s003.docx]

| **Reviewer comments** | **Response** |
| --- | --- |
| 1. The paper claims that knowledge translation approaches are useful in improving the uptake of evaluation in the implementation of complex health programs with multiple stakeholders. the study is useful to the discipline moving beyond research findings more commonly found in literature to evaluation co-production. | We thank the reviewer for their kind comments. |
| 2. The paper is placed within the growing field of the use of knowledge translation to increase uptake of evaluation findings. The authors do well to demonstrate the relevance of knowledge translation in evaluation work. The authors can improve by providing examples from the existing studies and describing their results and methodology briefly. | Thank you. We have attempted to do this in the introduction section by adding the following:  “In a case study assessing the role KT plays in the use of evaluations in foundations and non-governmental organisation, KT was reported to enhance use of evaluations by promoting understanding, facilitating decision-making, and improving program effectiveness(17)..” However, there are limited studies that have used a KT/iKT approach to improving evaluation use. |
| **Reviewer 2** |  |
| 1. The use of the qualitative approach method (semi-structured questionnaire) makes it difficult for one to conclude how the saturation phenomenon was achieved in these questionnaires and how we can say that the opinions being expressed represent fully the reality (link with the selection criteria that should be done within a strategy to cover all aspects that are presented in the objectives list but also avoid biases) | Thank you.  Reaching saturation was not a particular aim for this study. A key hallmark of qualitative research and the sociological perspective through which we view iKT, is that there are multiple realities. Because you cannot sample everyone’s realities, reaching saturation becomes impossible. The aim of the paper becomes about sharing the about the perceptions of the participants which are valid on their own because those are their realities. In addition, we sampled 3 different groups of participants to enrich what we’ve learnt from each group and promote representativeness. This doesn't negate the validity of our findings but highlights the specific context and participants' voices represented. |
| 2. It’s not clear how saturation was achieved in these semi-structured questionnaires given that half of the research participants dropped off during the study period and how we can say that the opinions being expressed represent fully the reality on the ground. | Thank you. This is unfortunately a limitation of the study and highlights a key issue that is important to consider in other iKT studies. The high staff turnover within the study sites meant that some of the participants who were involved in the iKT processes were not at their posts to implement their action plans/report on outcomes. Such a challenge affects the sustainability of the uptake. This is mentioned in the discussion:  “In addition, the high staff turnover meant that out of the 105 participants who attended the workshops, only 25 were still at their stations and could implement the action plans and report on them. Facility-in-charges who were not at the workshop were unaware of the action plans. This highlights issues with the spread of the evaluation findings amongst the stakeholders, thus presenting a barrier to sustained change. Future iKT approaches for similar contexts must consider how new stakeholders are updated.” |
| 3. At the grassroots level, we have the community health workers, who play a critical role in sensitisation and health education at the community level and are the critical link between the facility and the mothers (consumers of the service). why were they not considered part of the study? On the other hand, we have mothers who are consumers of free maternity services. I am grappling on why they were not part of the study. | Thank you for your comment. Indeed, the views of mothers and community health workers are important in improving health programs. However, when engaging with the stakeholders we reflected on the goal of our iKT activities which was to support uptake of evaluation findings at the policy-decision level. Mothers were included as participants in the process evaluation of Linda mama (study referenced in the paper) where their views and experiences as services users were captured.  However, the study this article describes sought to explore the usefulness of iKT in the uptake of evaluation findings by decision-makers at various policy levels to address the issues with the Linda mama implementation at the policy level. |
| 4. The study states the three counties as explained in the study area section, but in the findings, they have a new label; for example, Isiolo is a study site but given a label as County A, making it confusing. I is important to maintain uniformity and sticking to the original label to give the reader a simple time | Thank you for highlighting this. For anonymity purposes, we have removed identifying information like the county names from the methods and results section.  In the methods section, we have only mentioned that 3 counties were sampled and provided rationale for sampling.  In the results section, the counties are now identified as either County A, B or C |
| 5. Table 1 is too long. It’s advisable if they subdivide the table based on themes guided by the study objectives instead of counties; this will allow easy comparisons of issues across the three study sites. For example, if administrative issues cut across the three countries, this can make a difference. | Thank you for suggesting shortening Table 1. We understand your concern about the table's length. We have explored various ways to condense the table while preserving essential information. However, doing so would compromise the clarity and comprehensiveness of the data presented. However, if brevity is a significant concern, we are open to moving the table to the supplementary materials and refer to it in the results section. |
| 6. The discussion section needs to be strengthened by clearly stating the a) strengths and weaknesses of the study; b) strengths and weaknesses in relation to other studies that have been carried out c) discussion of important differences in results, meaning of the study, unanswered questions, and future research. | Thank you. We have included findings from other studies in relation to our findings in the discussion section. We have also addressed study strengths and weaknesses. |
| 7. The conclusion should provide a brief summary of the key findings, potential implications, and the way forward. | Thank you. The conclusion has now been revised to:  “Our findings report uptake of evaluation findings and suggest perceived improvements in the implementation of Linda Mama in 3 Kenyan counties through our iKT approach. However, these improvements were not uniform across and within the counties. Contextual factors such as the COVID-19 restrictions, lack of infrastructure and delayed reimbursement of funds hindered the full implementation of the evaluation findings. Overall, participants appreciated the value of the iKT approach and acknowledged its influence in supporting the use of the evaluation findings by promoting stakeholder engagement and providing a space for learning and sharing. This study contributes to the literature on utilising evaluation findings of complex, multi-stakeholder health programs by demonstrating the value of iKT approaches in supporting use. Although we report positive outcomes with this approach, iKT strategies can be challenging. Future approaches should consider the impact of context and how to navigate it and how best to sustain change, especially given a high turnover of stakeholders in decision-making positions.” |
| 8. It’s not clear from the abstract what qualitative techniques were used to collect and analyse the data for this study | Thank you. This has now been revised by adding:  “We used a case study approach employing qualitative approaches to describe our KT strategy and understand its impact on evaluation use. Data was collected through semi-structured in-depth interviews of participants (n=25) in three Kenyan counties following dissemination of the evaluation findings and co-production of action plans based on the evaluation.” |
