# Supplementary material for: Using knowledge translation to support the use of evaluation findings: A case study of the linda mama free maternity program in Kenya
Source: PLOS Glob Public Health. 2024 Dec 12;4(12):e0003961. doi: 10.1371/journal.pgph.0003961 (PMC11637383; doi:10.1371/journal.pgph.0003961)
Supplement: Attachment — Submitted filename: 02092024_Response to reviewers_PLOSGPH.docx [file pgph.0003961.s004.docx]

| Reviewer comment | Response |
| --- | --- |
| 1. Please include a complete copy of PLOS’ questionnaire on inclusivity in global research in your revised manuscript. | Thank you. This has now been included |
| 2. Please review your reference list to ensure that it is complete and correct. | Thank you. This has been done. |
| 3. Generally, I found this to be an interesting study but it is perhaps trying to demonstrate too many areas in one paper. My sense is that it is doing four things: 1. Report on the design of an evaluation; 2. Report on the outcome of an evaluation; 3. Show how a partnership approach to evaluation was utilised; 4. Show how an IKT approach formed the basis of an evaluation. The introduction and the conclusion are reasonably focused on the use of KT/IKT as an approach but otherwise the paper veered between all of these four things and potentially lost some focus as a result. | Thank you for your insightful comment. We understand the concern about the potential lack of focus as various elements of the evaluation have been integrated into the paper. The focus of the paper was to report on the use of an KT strategy to promote the uptake of evaluation findings.  To address the comment and clarify the focus of the study, we have:   A) **In the abstract, the following is stated:** “We used a case study design employing qualitative approaches to describe our KT strategy and its impact on evaluation use.” B) **In the introduction section, the following is stated:** “In this paper, we sought to contribute to this body of evidence by describing an iKT strategy for increasing the uptake of the findings from a process evaluation of a health program in Kenya and its impact. While the design of the evaluation, and its outcomes are discussed briefly to provide context, the primary focus of this paper remains on how the KT strategy facilitated the use of evaluation findings.” C) **In the results section: we added the following statement at the beginning to clarify that the outcomes presented here are the outcomes of the action plans that were implemented:** “To measure the impact of our iKT strategy, we assessed the extent to which the action plans that were co-developed with stakeholders had been implemented. Table S1 outlines the co-created action plans and progress on their implementation at the time of data collection.” D) **To the discussion section, we added the following:** “This study contributes to growing body of literature by offering insights into how deliberate integration of KT in evaluation processes can enhance the practical use of evaluation findings in practice settings. This findings from this study can guide other researchers undertaking process evaluations by highlighting opportunities and possible pitfalls” |
| 4. It would be useful to define KT when it is first introduced as a concept as there are myriad definitions and terms in existence. It appears the authors are using Strauss et al as their preferred reference point but that is not clear. Also, on page 3 they have referred to the purpose of KT as promoting research uptake. However, the purpose is somewhat broader than they have suggested i.e. to improve health, the provision of effective services, to enhance the use of research in practice, policy, guidance etc. and to potentially stop doing what is ineffective and wasteful. In essence, it bridges the gap between research knowledge and its application in practice. | Thank you. The definition of KT has now been provided (according to Straus’s definition. We have also clarified the broader purpose of KT.  “KT can be defined as the “methods for closing the gaps from knowledge to practice” (13). Its overall purpose is to promote research uptake during clinical and policy decision-making to provide effective health services and polices for improved health outcomes (14, 15). |
| 5. It is not clear if the research team used KT/IKT as a tool within the evaluation framework or simply saw it as an approach. An obvious limitation of the study would be a failure/lack of integration of the KT approach into the evaluation design. The approach as described seems to be very much focused on KT in mobilising findings when in fact KT starts much earlier in the process. | Thank you for your comment. The KT approach was indeed integrated into the evaluation design. Thus, it was not only a tool used for mobilising findings but was an integral part of the entire evaluation process. Specifically, we employed KT at the three steps mentioned in the methods section:   a) Co-design: stakeholders were involved in the design and conduct of the evaluation b) Co-production of action plans based on the findings of the evaluation. This step was used to translate the findings of the evaluation into actionable solutions to address the issues highlighted in the evaluation c) Evaluation on the implementation of the evaluation-informed action plans to assess the success of our KT/iKT approach   We have attempted to articulate this better by adding the following:  **To the beginning of the methods section:** “Our iKT approach to improving the implementation of the Linda Mama program in three Kenyan counties embedded KT principles at every stage of the evaluation and involved three steps..”  **To the beginning of the results section, we added:** “The inclusion of stakeholders from the outset of the evaluation process ensured that the evaluation was aligned to their needs and priorities which in turn facilitated the co-development of contextually appropriate action plans.”  **To the discussion section, we added the following:**  “In our study, researchers engaged with stakeholders throughout the evaluation process from the design of a process evaluation, including setting objectives and the overall design (sample, population, etc.) to the co-development of evaluation informed action plans.”  **In the conclusion, we have added the following:**  “When embedded throughout the evaluation, KT can significantly enhance the use and implementation of evaluation findings.” |
| 6. A minor point of semantics but on page 4 paragraph 3 refers to a ‘free maternity policy’, I presume they mean a ‘policy providing free maternity health care’. | Thank you. This has been revised to “policy providing free maternity care” and in the subsequent sentences in the paragraph, referred to as “the policy” or “Linda mama” |
| 7. On page 5 the heading ‘co-research’ did not seem to reflect the activity usually associated with the term. Co-research usually suggests that co-researchers have been part of the research team throughout the project but in this study their role seems to have been linked to two key activities pre and post implementation of the Linda Mama programme. Step 1 states that stakeholders were asked to identify key research questions. Presumably this activity may have generated a number of questions but it is not clear if they were all used or whether there was a process for selecting those that were deemed most relevant or important. | Thank you for your feedback regarding the term “co-research”.  In the revised manuscript, I have reconsidered the use of the term 'co-research' to better reflect the level of stakeholder involvement in the research process. While stakeholders played a significant role in shaping the research, their involvement was concentrated in specific stages (design of the evaluation and action plan development) rather than being continuous throughout the entire project (due to time constraints), as the term 'co-research' might imply. We have therefore updated this to “co-design” |
| 8. Were the participants in the study and the stakeholders references throughout the paper the same people or are they different groups? It is not clear if there is a distinction or whether the study participants were actively engaged with the stakeholders who were implementing the evaluation findings. Similarly, on page 85, it was unclear who the stakeholders were and whether they included some of those identified in the previous section as being barriers to implementation. | Thank you for pointing this out. The stakeholders were the participants in the study as they were the implementers of the action plans.  This has been clarified as below:  In page 6 & 7: the word stakeholder is used. Where we wanted to distinguish the implementing stakeholders from the broader group of stakeholders, we use the term “implementing stakeholders” |
| 9. The results section did not seem to be focused sufficiently on which aspects of IKT worked. A series of quotes was included which, whilst interesting, did not drill down into which of the technical aspects of IKT worked. There was also very little in relation to the impact and sustainability aspects of KT. It was not clear if the IKT elements of the evaluation activity actually provided any training or information about IKT for the participants, how it added value to their implementation process and how to use IKT to aid more effective information sharing and implementation. The use of one or more KT theories, models or frameworks may have been helpful to the researchers in this context but there is no indication that this was a consideration for them. | Thank you for your comment. We have revised the results section to clearly articulate these points.  As part of this revision, the results section is now structured as below   1. Impact of the iKT strategy 2. Key aspects of the strategy that worked 3. The challenges faced   We believe structuring the results section in this way makes our findings easier to understand.  We did not set out to train stakeholders in the iKT approach per se but rather to integrate iKT principles into the evaluation process itself. The goal was to create a collaborative environment where stakeholders could actively participate in the design, interpretation, and application of evaluation findings. While this approach was effective in achieving our objectives, future research could explore the added value of providing explicit iKT training to stakeholders to further enhance their capacity for knowledge translation especially given that one challenge we identified is the poor spread of evaluation findings amongst the stakeholders.  In this study, we focused on a pragmatic application of iKT and participatory evaluation principles focusing on engagement and collaboration with stakeholders. While we did not use KT theories, models or frameworks, we drew upon Smits and Champagne’s model of practical participatory evaluations to inform our strategy & make sense of our findings. This has now been clarified in the methods and discussion sections |
| 10. In the discussion the authors mention the participants wish to have the researchers engaged to a greater degree to foster sustainability but the researchers role was limited to evidence generation and they could not maintain continuous engagement. This should be cited as a limitation of the study as a key component of KT is to establish who is engaged in the KT process (i.e. stakeholders), how and when and how the activity will be sustained. The research teams lack of capacity and not planning for who would maintain the KT activity is something for the authors to reflect on in terms of using (or partially adopting) a KT approach. | Thank you for this insightful comment. We agree that a limitation of the study is the research team's inability to maintain continuous engagement throughout the KT process, which could have impacted the sustainability of the knowledge translation activities. While our role as researchers was primarily in evidence generation and conceptualising the KT approach, we partnered with a development partner (ThinkWell) to support implementation by providing technical assistance and maintaining ongoing engagement.  We have acknowledged this limitation in the discussion by revising to:  “A key challenge with our KT strategy was its sustainability. While the research team was highly involved initially, we could not continue this high-level engagement during the implementation refinement stage. In this study, the researchers' main roles were evidence generation through the process evaluation, dissemination of these findings and facilitating the co-development of action plans. To support implementation of the action plans, we collaborated with a partner (ThinkWell) to provide technical assistance and support to the implementers. However, the study participants expected continuous engagement with the researchers over the implementation duration, which was not sustainable due to resource constraints. Future studies should consider how best to plan for sustainability earlier in the KT process.” |
| **Reviewer 4:** |  |
| 11. Data – plural (throughout manuscript) | Thank you. This has been adressed |
| 12. Abstract describes: case study approach using qualitative approach. In another section “participatory approach” (used twice) and “KT approach”. Can the word “approach” be limited? | Thank you. This has been addressed. To describe KT, we have used the work “strategy”. For case study, we have changed that to “design” |
| 13. I found the word approach confusing because it was used to describe different things in the same paragraph and sentence overall. The revisions added in the word “approaches” | Thank you. We have revised this as in the comment above this. |
| 14. Page 3 – “while the literature in both fields has grown” | Thank you. This has been clarified further to “the literature on both KT and evaluation use..” |
| 15. Some grammar issues with added text (page 3) – | Thank you. This has been revised to the best of our knowledge |
| 16. Page 26 – Discussion – where the revision was made – it has been reported to increase what type of use? | Thank you. We have clarified this to:  “..reported to lead to greater use of evaluation findings” |
| 17. Revisions need to be copyedited for grammatical issues | Thank you. This has now been done to the best of our ability. |
| 18. Re: addressing limitations –might the the depth of information from the interviews provided preliminary evidence that might be explored in different ways (using quantitative data, and/or further qualitative data gathering) in the future? | Thank you for highlighting the potential to build on our qualitative findings. While our aim was to capture the nuanced perceptions of the stakeholders, we agree that future research can benefit from incorporating quantitative methods to enrich our understanding of the KT mechanisms at play. For example, by using surveys, we can quantify stakeholders’ preferences for engaging in KT so as to support development of KT/iKT strategies to promote use of evaluation findings. |
| 19. While the comments have been addressed – identifying the benefit of this work to add to the literature, and support integration between KT and evaluation use would be beneficial. It might be possible to add references to this at the beginning of the discussion – this emphasizes why the sample was chosen for the interviews | Thank you.  We believe the benefit of this study to the growing literature is to offer insights into the benefit of integrating KT into evaluation processes to improve uptake of evaluation findings. This case study provides a practical example of this, highlighting the facilitators and barriers of KT/iKT in evaluations.  We have added the following to the discussion (pg14-15):  “This study contributes to growing body of literature by offering insights into how deliberate integration of KT in evaluation processes can enhance the practical use of evaluation findings in practice settings. This findings from this study can guide other researchers undertaking process evaluations by highlighting opportunities and possible pitfalls.”  The sample was chosen based solely on their involvement in both the KT process and in implementing the action plans. This targeted sampling allowed us to explore the effectiveness of KT in promoting uptake of evaluation findings, thereby contributing empirical evidence to the literature on KT and evaluation use. |
| 20. How were the qualitative data analyzed. This is still not completely clear. What strategy was used for this? When I read the description of the analysis I am not totally sure whether the data were coded based on the interview guide? (e.g., the 3 key issues of investigation?)? You also refer to a thematic framework that was developed by the FHG and SO (is FHG – a person?) | Thank you. Yes, FHG is the lead author.  **To clarify the process used, we have added the following to the methods section (pg 8):**  “The transcripts were read and reread (by FHG and SO) to familiarise ourselves with the data. We began our analysis by conducting open coding where key concepts and patterns were identified through and inductive process. Subsequent rounds of coding were more focused and based on the three key issues of investigation highlighted in the interview guide. These key issues served as our primary themes. Any emerging sub-themes from the primary themes were noted. After these initial rounds of coding, we developed a coding framework which was iteratively refined as coding progressed. All predefined themes and any other additional themes were captured within the framework. To ensure rigour during the coding process, coding was conducted by 2 coders (FHG and SO) independently. Both coders met regularly to discuss the coding framework and codes. All transcripts were coded using NVIVO qualitative data analysis software (QSR International Pty Ltd. Version 12, 2014).” |
| 21. The qualitative data findings do not seem to reveal the thematic framework. Are there themes and do they relate to the 3 key issues of investigation? | Thank you for your comment. I will clarify how the 2 main themes were derived and how they relate to the 3 key issues of investigation:  Theme 1: Use of evaluation findings: This theme focused on the influence of the KT strategy on the specific use of the evaluation findings through implementation of action plans. This theme is presented in the results section under the heading: impact of the iKT strategy  Theme 2: Influence of the iKT approach  This theme presents the perceptions of how the KT strategy facilitated the use of evaluation findings. This is under the following sub-headings:   - Key aspects of the iKT strategy that worked and - Challenges to the iKT strategy |
| 22. I agree with reviewer 2 that the Table 2 is extremely lengthy and hard to follow. Is it possible to add Table 2 to an addendum, but shorten table 2 for the manuscript to include the essential elements? (i.e., make it shorter and easier for a reader to take in on less space)? | Thank you.  After consideration, Table 2 has now been moved to supplementary file 1 (Table S1) |
