# Supplementary material for: Using knowledge translation to support the use of evaluation findings: A case study of the linda mama free maternity program in Kenya
Source: PLOS Glob Public Health. 2024 Dec 12;4(12):e0003961. doi: 10.1371/journal.pgph.0003961 (PMC11637383; doi:10.1371/journal.pgph.0003961)
Supplement: Attachment — Submitted filename: 28102024_Response to reviewers_PLOS GPH.docx [file pgph.0003961.s005.docx]

| Reviewer comment | Response |
| --- | --- |
| 1. Proofread entire document | Thank you. We have proofread the manuscript to the best of our ability and made the necessary changes |
| 2. Please include the following request in the decision letter and ping me with follow-up. | This has been included as one of the uploaded documents under supporting information: |
